# Broad Neutralizing Antibodies Against SARS-CoV-2: Current Progress and Engineering Strategies

**DOI:** 10.3390/v18060642

**Published:** 2026-06-03

**Authors:** Tianrong Jia, Zehong Huang, Ningshao Xia, Quan Yuan

**Affiliations:** 1 State Key Laboratory of Vaccines for Infectious Diseases, Xiang An Biomedicine Laboratory, School of Public Health, School of Life Sciences, Xiamen University, Xiamen 361102, China; jiatianrong@163.com (T.J.); nsxia@xmu.edu.cn (N.X.); 2 National Institute of Diagnostics and Vaccine Development in Infectious Diseases, Collaborative Innovation Center of Biologic Products, National Innovation Platform for Industry-Education Integration in Vaccine Research, Xiamen University, Xiamen 361102, China

**Keywords:** SARS-CoV-2, broadly neutralizing antibodies, conserved epitopes, nanobodies, antibody cocktails, bispecific antibodies, artificial intelligence

## Abstract

The high-frequency mutation characteristics of SARS-CoV-2 have posed formidable challenges to the development of vaccines and therapeutic agents. Neutralizing antibodies, which serve as effective tools for prevention and control, have undergone continuous updates and iterations in response to viral mutations. This article provides a comprehensive review of researchers’ efforts to achieve both high neutralizing potency and high mutation tolerance in SARS-CoV-2–targeting neutralizing antibodies. Building on the characteristics of conventional antibodies directed against distinct epitopes on the S protein, it further discusses the research on nanobodies, antibody cocktails, multi-specific antibodies, and other antibody formats and engineering approaches, including artificial intelligence–enabled optimization. Each antibody-based strategy targeting SARS-CoV-2 has its own distinctive advantages and potential applications, providing an integrated perspective to support the continued development of antiviral neutralizing antibodies.

## 1. Introduction

The COVID-19 pandemic has inflicted substantial public health damage worldwide over the past several years. As a key countermeasure beyond vaccines, monoclonal antibodies (mAbs) have demonstrated irreplaceable value in the treatment and prevention of SARS-CoV-2–related disease.

The Spike (S) protein is critical for host cell recognition by SARS-CoV-2 and for initiating the infection process. Viral infection of cells is accomplished through a series of proteolytic cleavages and conformational changes involving multiple domains of the S protein, including the receptor-binding domain (RBD), N-terminal domain (NTD), subdomain 1 (SD1), fusion peptide (FP), and stem helix (SH). This process encompasses a series of biological events, such as host ACE2 binding to the RBD, dissociation of the S1 and S2 subunits, proteolytic cleavage at the S1/S2 and S2′ sites, and conformational changes in the S2 subunit [1,2,3,4]. The S protein is a metastable trimeric glycosylated fusion protein comprising multiple domains; epitopes that are highly accessible, strongly immunogenic, and highly conserved can serve as key targets for the screening and development of neutralizing antibodies (nAb) [5,6,7]. However, the continual emergence of escape variants driven by the high mutability of SARS-CoV-2 has become a major constraint on the clinical efficacy of mAbs.

Accordingly, during the development of nAbs targeting SARS-CoV-2, antibody neutralizing activity, mutational tolerance, and in vivo protective efficacy have become major considerations. Researchers have employed a variety of technical approaches to screen and engineer antibodies with high neutralizing potency and mutational tolerance. The nAbs selected based on the epitope conservatism of the S protein exhibit stronger tolerance to escape mutations. Due to their small molecular size, nanobodies can more readily access cryptic epitopes that are difficult for conventional antibodies to reach. Antibody cocktails broaden the neutralization spectrum of the component antibodies; moreover, rationally designed cocktails may exhibit synergistic enhancement effects that are not present in the individual components. Bi-/multi-specific antibody technologies have also been applied to research on the prevention and treatment of COVID-19. Multi-specific antibodies enable more flexible integration of the strengths of different antibodies; furthermore, conversion into a multi-specific format can introduce new neutralization mechanisms, thereby further enhancing nAb function. Isotype switching, multivalency engineering, and Fc engineering can further optimize antibody functional performance. In addition, artificial intelligence (AI)-based antibody engineering has played an important role in the development of SARS-CoV-2 nAbs from multiple dimensions, including antibody optimization and de novo design.

Here, we provide a comprehensive review of the characteristics and advantages of representative conserved-epitope-based broadly nAbs, nanobodies, antibody cocktails, multispecific antibodies, and AI-engineered antibodies, thereby offering a relatively comprehensive overview of development strategies for antibodies targeting SARS-CoV-2.

## 2. Broadly Neutralizing Antibodies

### 2.1. Broadly nAbs Target RBD

**Figure 1:**
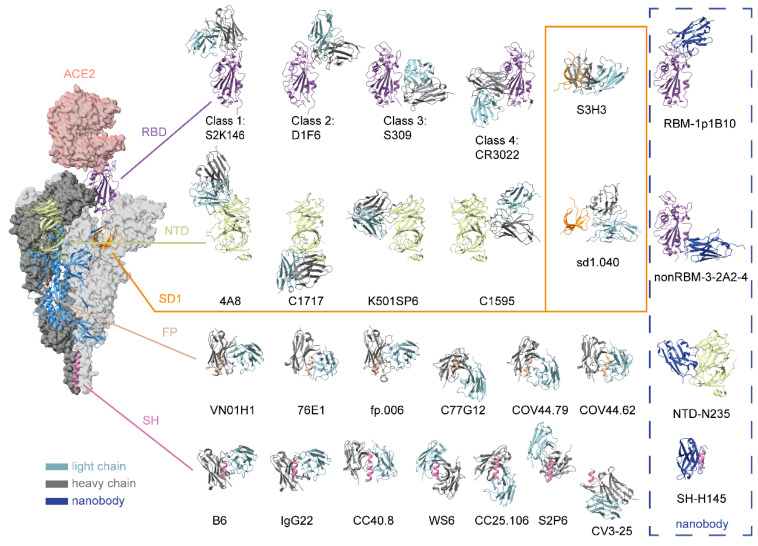
Representative antibodies and nanobodies targeting the SARS-CoV-2 spike. Antibody/antigen complex structures are from the Protein Data Bank (PDB). The spike trimer (PDB: 7N1Q) is shown using a bimodal representation. Two of the protomers are displayed with their molecular surfaces in dark and grey, the third protomer is illustrated as a cartoon. Representative binding patterns of neutralizing antibodies targeting various epitopes on the S protein, RBD-ACE2 (6M0J), S2K146 (7TAT), D1F6 (8ZBY), S309 (7R6W), CR3022 (6W41), 4A8 (7C2L), C1717 (7UAR), K501SP6 (9FJK), C1595 (9BJ3), S3H3 (7WK8), sd1.040 (8D48), VN01H1 (7SKZ), 76E1 (7X9E), fp.006 (8D47), C77G12 (7U0A), COV44.79 (8DAO), COV44.62 (8D36), B6 (7M53), IgG22 (7S3N), CC40.8 (7SJS), WS6 (7TCQ), CC25.106 (8DGU), S2P6 (7RNJ), CV3-25 (7RAQ), 1p1B10 (9IQP), 3-2A2-4 (7X2L), N235 (8JVA), H145 (9LDS). RBD, receptor-binding domain. NTD, N-terminal domain. SD1, subdomain 1. FP, fusion peptide. SH, stem helix.

The mAbs targeting the SARS-CoV-2 Spike recognize a diverse array of epitopes, covering various domains along the full length of the Spike protein (Figure 1 and Table A1). Among them, the RBD serves as the primary target for nAbs, and multiple approved antibodies are directed against the RBD. However, as one of the regions most enriched in mutational epitopes, nAbs targeting the RBD are also most susceptible to viral escape [8,9]. Based on the epitope location and binding mode of nAbs against the RBD, these antibodies can be further classified into multiple types. Barnes class 1/2 nAbs show a higher frequency of escape mutations at their epitopes, whereas class 3/4 antibodies have relatively more conserved epitopes [10]. Barnes class 1 and 2 antibodies are more readily escaped by the virus; however, benefiting from high epitope immunogenicity, these nAbs are numerically abundant and exhibit high neutralizing activity. Furthermore, epitopes of nAbs targeting the RBD are typically conformational epitopes, and after multiple rounds of screening, broadly nAbs with conserved epitope residues can still be obtained. For instance, the class 1 antibody BA7535 recognizes a conserved epitope on the RBD composed of aa 415/420/421/475/487/489/493, which avoids most mutation hotspots and can effectively neutralize Omicron sublineages such as BA.5, BF.7, XBB.1, XBB.1.5, and EG.5 [11]. Similar class 1 antibodies include 17T2, 87G7, 10-5B, and AZD3152 [12,13,14,15], which require conserved key residues for binding, enabling them to retain neutralizing activity against multiple variants. Among them, AZD3152 has completed Phase III clinical studies and has submitted a marketing authorization application to the European Medicines Agency (EMA) [16]. Some class 1 antibodies can functionally mimic ACE2 in their neutralizing mechanism, causing dissociation of the S1 subunit and disrupting the preS protein conformation. For example, S2K146 has 18 of its 24 epitope residues overlapping with the ACE2 binding site, mimicking ACE2 through electrostatic interactions and shape complementarity. It exhibits neutralizing activity against Omicron BA.1.1, BA.2, BA.3, BA.2.12.1, BA.4/5, XBB.1.5, EG.5.1, and HK.3 [17,18,19,20]. GAR05, ZCP3B4, P2-1B1, P4J15, KXD03, and VIR-7229 [21,22,23,24,25,26] employ similar neutralizing mechanisms to S2K146, and their critical binding residues are also relatively conserved. The class 1 monoclonal antibody BD55-1205, selected through deep mutational scanning (DMS)-based prediction of escape epitopes and also exhibiting ACE2-mimicking characteristics, demonstrates potent broad-spectrum neutralizing activity. It achieves broad neutralization against Omicron HK.3.1, JN.1, and KP.3 via its broad binding to the epitope involving aa 455, 456, 473, and 475 [27]. Another broadly neutralizing class 1 antibody, P5-1C8, maintains neutralizing activity against the Omicron JN.1 despite reduced binding affinity by mediating inter-S protein bridging and inducing aggregation [28]. Class 2 antibody epitopes are prone to mutation and are highly susceptible to escape by new variants. Some class 2 antibodies with conserved key residues at conformational epitopes can maintain neutralizing activity against certain Omicron sublineages, such as P5S-2A9, D1F6, A19-46.1, and COV2-2196 [8,23,29,30].

Class 3 and 4 antibody epitopes are located distant from the ACE2 binding site. The class 3 antibody S309 restricts the RBD from adopting an “up” conformation, and its binding to the S protein creates a spatial barrier that prevents ACE2 binding by obstructing S protein conformational rearrangement [31]. S309 exhibits broad neutralizing activity against variants including Omicron BA.1.1, BA.2, BA.2.75, BA.4/5, BQ.1.1, XBB.1.5, EG.5.1, HK.3, and JN.1 [20,32]. Other broadly nAbs with mechanisms of action similar to S309 include SP1-77 and SW186 [33,34]. The class 3 antibody LY-CoV1404 (bebtelovimab) has an epitope that extends into part of the RBM and exerts neutralizing effects by obstructing ACE2 binding [35]. BA7208, S2X324, P2S-2E9, and 6-2C share binding patterns similar to LY-CoV1404, and all can broadly neutralize multiple Omicron sublineages [14,20,23,32,36,37]. The class 3 antibody 1G11 does not directly interfere with RBD-ACE2 interaction; however, it can induce “head-to-head” aggregation of S protein trimers, creating steric hindrance or occluding the receptor binding site [32]. Among the antibodies mentioned above, both S309 and LY-CoV1404 received FDA EUA but were revoked due to the impact of non-susceptible variants [38,39].

The class 4 antibody CR3022, derived from a SARS-CoV patient, binds to an epitope that does not overlap with the ACE2 binding interface [40]; however, this antibody may exert its biological function by inducing dissociation of S protein trimers [41]. ADG20, Ab246, and DH1047 [30,42,43,44] have epitopes that partially overlap with CR3022 and also belong to the class 4 category; their neutralizing activity against certain variants is reduced. ADG20 completed Phase II/III clinical studies in 2022, but its clinical development was halted due to the impact of immune evasive variants [45]. The class 4 antibody AB2-122, identified via screening in an engineered mouse model, utilizes dual aromatic residues within its HC-CDR3 to recognize a hydrophobic epitope on the Omicron-mutant-remodeled RBD. It achieves broad neutralization against multiple Omicron sublineages (including NB.1.8.1 and XFG) by blocking ACE2 binding [46].

In addition to class 1–4 antibodies, there are also antibodies targeting more cryptic epitopes on the RBD that similarly exhibit potent neutralizing activity and broad specificity. S2H97 binds to a cryptic conserved epitope on the RBD without interfering with ACE2-RBD interaction, and it can induce S protein refolding into a post-fusion state [47], thereby exerting broad-spectrum neutralizing effects against SARS-CoV and SARS-CoV-2 variants including Omicron BA.1, BA.2, XBB.1.5, EG.5.1, HK.3, JN.1, and others [20]. XMA09 and C68 [48,49], which have epitopes similar to that of S2H97, likewise demonstrate significant neutralizing activity against Omicron sublineages and SARS-CoV. GAR12 binds to an epitope located between those of S309 and S2H97, and exhibits neutralizing activity against BA.5 and earlier Omicron sublineages [21]. ION_300 binds to a unique region on another side of the RBM that is occluded by the NTD of adjacent S1 when the RBD is in the “down” conformation [50]; it can effectively neutralize SARS-CoV and SARS-CoV-2 variants BA.2, XBB.1.5, EG.5.1, HK.3, and JN.1 [20] (Figure 1).

### 2.2. Broadly nAbs Target NTD

Most antibodies targeting the NTD use an antigenic supersite as their epitope, which is located on the periphery of the S protein and represents the largest non-glycosylated surface on the NTD, composed of the N1, N3, and N5 loops. However, this supersite is prone to mutations, with deletions such as aa 69–70del and 144del (Alpha, B.1.1.7), and aa 242–244del and R246I (Beta, B.1.351) all located at the NTD supersite [51,52]. Antibodies of this class include 5–7, C1717, C1520, and C1791 [53,54,55]. Additionally, some NTD nAbs bind to unique epitopes on the N1/N2 loops, competing with ACE2 and blocking S1 shedding; however, insertions or deletions in the N1/N2 loops can cause such antibodies to lose neutralizing activity [56]. If the binding epitope of nAbs targeting the NTD can involve other regions, the breadth of their binding and neutralizing activity may be enhanced. The critical residues of the K501SP6 epitope are aa 122–123, 225, and 163–170; this epitope is relatively conserved in most variants, and its binding involves both the NTD and SD1, forming a cross-domain epitope that can cross-neutralize multiple variants including Omicron BQ.1.1.20, XBB.1, and JN.1 [57]. The C1596, targeting non-antigenic supersites of the NTD, can recognize a quaternary epitope containing the NTD, RBD, and SD1, and can cross-neutralize Omicron XBB.1.5, BA.2.86, and JN.1 [58] (Figure 1).

### 2.3. Broadly nAbs Target SD1

Several reported nAbs targeting SD1 exhibit broad-spectrum neutralizing effects. S3H3 binds to an epitope on SD1 comprising aa 323–324, 532–537, 554–556, and 581–584 [59]; its neutralizing mechanism may involve inducing a conformational change in the S protein that locks the RBD in a transitional state between “up” and “down” conformations [60]. S3H3 neutralizes Omicron XBB.1, XBB.1.5, EG.5.1, and HK.3 [61,62]. However, the E554 mutation present in BA.2.86 and JN.1 abrogates its neutralizing activity [62,63,64]. P008_60 primarily binds to the L3 loop of SD1 while also interacting with the L4 and L5 loops and the glycan at N331; N-linked glycosylation at N331 significantly reduces its neutralizing activity [65]. Sd1.040 targets SD1 and recognizes an epitope formed by SD1 aa 554–562 and 577–581, as well as RBD aa 520–524. It does not block ACE2 binding but potently inhibits ACE2-induced conformational changes in the S protein, enabling cross-neutralization of variants including Omicron BA.1, BA.2, BA.2.75, BA.2.75.2, and BA.4/5 [66]. The key binding residues for C68.59 are aa 554, 558, 577, 583, and 585. Upon binding SD1, it may trigger a conformational change that indirectly affects RBD-ACE2 interaction and leads to a marked decrease in the structural stability of the S2 subunit [67]. MO11 binds to a conserved epitope on SD1 located adjacent to the N331 glycan, connecting the N-terminal (aa 529–538, 552–555) and C-terminal (aa 322–332, 579–582) segments of SD1. It does not affect S protein binding to ACE2 and broadly neutralizes multiple variants including Omicron BA.1, BA.2, BA.2.75, BA.5, BQ.1.1, XBB.1.5, XBB.1.16, and EG.5.1 [68] (Figure 1).

### 2.4. Broadly nAbs Target FP

Transmembrane serine protease 2 (TMPRSS2) recognizes the R815 epitope on S2, cleaves the S protein, and exposes the FP, thereby triggering membrane fusion. As a functional epitope essential for viral infection, the FP sequence is relatively conserved and represents one of the ideal targets for broadly nAbs [69]. The mechanism of action for FP-targeting antibodies is relatively uniform; their binding epitopes are typically exposed after ACE2 engages the S protein, and they exert neutralizing effects by hindering S protein cleavage and inhibiting the membrane fusion process. The reported antibodies of this class also exhibit considerable convergence in their binding epitopes. For instance, 76E1, which binds to residues 809–833, exhibits neutralizing activity against both human α- and β-coronaviruses [70]. C20.119, which shares a similar binding epitope, can neutralize BA.1, BA.2, BA.5, BQ.1.1, XBB, XBB.1.5, and SARS-CoV [71]. Antibodies recognizing epitopes within the range of residues 813–825, such as COV44-62, COV44-79, VN01H1, VP12E7, C77G12, fp.006, and fp.007, are capable of effectively neutralizing Omicron sublineages, including BA.1, BA.2, and BA.4/5, among others [66,72,73] (Figure 1).

### 2.5. Broadly nAbs Target SH

The sequence of the SH epitope is highly conserved; therefore, antibodies targeting SH generally possess promising broad-spectrum potential. Representative antibodies such as S2P6, CV3-25, CC40.8, 7B2, WS6, S2-4D/S2-5D/S2-8D/S2-4A, 1249A8, hr2.016, and CC25.106/CC68.109/CC99.103 primarily recognize overlapping epitopes within the region of residues 1131–1171. They can neutralize early SARS-CoV-2 VOCs, certain Omicron sublineages, and even other coronaviruses such as SARS-CoV, MERS-CoV, and HCoV-OC43 by either blocking conformational transitions or inhibiting virus-host membrane fusion [74,75,76,77,78,79,80,81,82,83,84,85,86]. Among these, some antibodies additionally demonstrate a direct interference with the post-fusion transition. For example, CV3-25 prevents the S protein from adopting a fusion-active state through conformational clash [77], while 1249A8 blocks six-helix bundle formation by “capping” the SH peptide [84]. Furthermore, the SH antibody repertoire derived from IGHV1-46/IGHV3-23 germlines exhibit strong public immunological features. Their critical binding residues, F1148, L1152, and F1156, are highly conserved among β-coronaviruses, forming the structural basis for their broad reactivity [86]. However, despite the conservation of the SH epitope, obtaining potent nAbs remains challenging. Some SH antibodies, such as 28D9, 1.6C7, B6, and IgG22, can bind to the S proteins of multiple coronaviruses but fail to neutralize SARS-CoV-2 due to insufficient affinity or limited epitope accessibility, suggesting that their broad neutralization capacity could be further optimized through affinity maturation in the future [87,88,89] (Figure 1).

### 2.6. Other nAbs Target S2

Due to issues such as immunogenicity or accessibility, nAbs targeting other epitopes within the S2 subunit are relatively rare. Among them, 3D1 targets a six-amino-acid peptide segment within the heptad repeat 1 (HR1) domain. This epitope is only exposed during the pre-fusion hairpin intermediate state of membrane fusion. 3D1 effectively neutralizes SARS-CoV-2 and SARS-CoV but is evaded by the Omicron Q954H mutation [90]. RAY53 is a nAb targeting the apex of the S2 subunit, spanning the HR1 and central helix (CH) regions. It exhibits weak neutralizing activity against SARS-CoV-2 and MERS-CoV but shows no neutralizing activity against SARS-CoV or Omicron [91]. hMab5.17 targets a highly conserved epitope (aa 1164–1172) and demonstrates neutralizing activity against SARS-CoV and early SARS-CoV-2 VOCs [92].

## 3. Nanobodies

Nanobodies, primarily derived from the variable domains of camelid heavy-chain-only antibodies (VHHs) or shark immunoglobulin new antigen receptors (VNARs), are single-domain antibodies. They offer advantages such as small molecular size, structural stability, ease of access to cryptic epitopes, and amenability to engineering [93,94], thereby demonstrating unique potential in the development of broadly neutralizing antibodies against coronaviruses. Their primary limitation is a relatively short in vivo half-life; however, constructing multivalent or multispecific antibodies can significantly improve their persistence and enhance their therapeutic potential.

VHHs from camelids represent the minimal antigen-binding unit. Their breadth similarly depends on epitope conservation. However, due to their small size, they can more readily target cryptic epitopes that are difficult for conventional antibodies to access. In some cases, the small molecular format can even enhance neutralizing activity, as seen with VN01H1 and C77G12, whose single-chain variable fragment (scFv) formats outperform their full-length IgG counterparts [73]. Numerous VHHs have demonstrated broad neutralizing capabilities against SARS-CoV-2 variants: for instance, Nanosota-9 and Ma16B06 can bind to RBD in both “up” and “down” conformations and potently neutralize Omicron sublineages [95,96]; 1p1B10, Tnb04-1, Nb1, Nb2, W25, and Nb4 target conserved RBD epitopes and the first four can neutralize variants including Omicron JN.1 [97,98,99,100,101]; 3-2A2-4 exerts broad neutralization by inhibiting the transition of RBD from “down” to “up” [102]; the engineered nanobody C5G2 uses its CDR3 loop to simultaneously engage the RBD and the adjacent NTD, and employs FR2-mediated steric hindrance to inhibit ACE2 binding [103]; IBT-CoV144 neutralizes by crosslinking two S proteins [104]; N235 recognizes a conserved cryptic epitope on the NTD and likely neutralizes multiple Omicron sublineages by interfering with the adjacent RBD and inducing S1 shedding [105]; H17 and H145 target the linear stem-helix (SH) epitope (aa 1139–1152), are effective against Omicron JN.1, KP.3, and SARS-CoV, and retain inhibitory activity even post-fusion as their binding is unaffected by acidic pH [106]. R3DC23 binds to the juxtamembrane region of HR2 (residues 1192–1206), recognizing a quaternary epitope formed by the trimeric HR2 coiled-coil, thereby achieving broad neutralization against SARS-CoV and prevalent SARS-CoV-2 variants [107].

Additionally, shark-derived VNARs constitute another important class of nanobodies. Among these, 79C11 targets a highly conserved region within the S2-HR1 domain, neutralizing SARS-CoV, Omicron JN.1, and KP.2, and can be administered intranasally to prevent XBB infection [108]; the nurse shark-derived S2A9 targets the S2 subunit and exhibits neutralizing activity against Omicron BA.1, BA.2, BA.4/5 [109]. Table 1 summarizes the representative SARS-CoV-2-specific nanobodies discussed above, including their origins, targets, binding affinities, neutralizing activities against Omicron sublineages, and neutralization mechanisms.

## 4. Antibody Cocktails

Acquiring mAbs that concurrently exhibit high potency and broad neutralizing is challenging, and antibodies targeting a single epitope still face a high risk of viral escape. Consequently, constructing antibody cocktails targeting non-overlapping epitopes has emerged as a key strategy to enhance coverage breadth, reduce escape risk, and achieve synergistic effects. Firstly, cocktails can expand the neutralizing spectrum through complementarity of their components. For example, while individual components of AZD7442 cannot comprehensively cover multiple Omicron sublineages, the combination achieves complementary neutralization [18]. AZD7442 previously received an FDA Emergency Use Authorization (EUA) in 2021. However, in 2024, the EUA for this antibody cocktail was revoked due to the impact of non-susceptible variants [113]. Similarly, the triple-antibody combinations IMM-BCP-01 and EGH rely on component complementarity to broaden their neutralizing range [114,115]. IMM-BCP-01 advanced to Phase I clinical studies in 2022, but its clinical development has since been terminated [116]. Secondly, antibody combinations targeting non-overlapping RBD epitopes can generate significant synergy. For instance, B1-182.1 and A19-46.1 significantly enhance neutralization against Omicron by inducing a triple-RBD-up conformation and “cooperative trapping” [8]; XMA01, XMA04, and XMA09 can form an inter-antibody interaction network on the same RBD to enhance overall binding stability [48]; H014 and HB27 produce an allosteric synergistic effect through conformation induction [117]. Moreover, cocktails targeting non-overlapping epitopes generally possess stronger resistance to viral escape. REGN-COV-2 is less prone to escape compared to competitive or partially competitive combinations [118]; SA55 and SA58 achieve robust, broad-spectrum neutralization against multiple SARS-CoV-2 variants by improving epitope coverage and the genetic barrier to escape [119]; Long-term serial passage experiments also demonstrate the superior resilience of high-coverage, non-overlapping combinations. The combinations 9E12+10D4+2G1 and 7B9-9D11+2G1 showed no breakthrough after 30 passages, whereas the partially overlapping epitope combination 10D4-8G4+2G1 was escaped within just 5 passages [120]. Among the antibodies above, REGN-COV-2 received FDA EUA in 2020, but its authorization was revoked in 2024 due to non-susceptible variants [121]. SA55, as a monotherapy, has entered Phase II clinical studies [122]. Combining antibodies across different S protein subunits, particularly S1 and S2, represents another valuable combinatorial direction. S1 antibodies typically exhibit higher neutralizing potency, while S2 antibodies offer broader coverage due to conserved targets. This pairing enables functional complementation and, under certain conditions, mechanistic synergy. The S2’/FP-targeting antibody 76E1 can be combined with ACE2-binding or ACE2-mimicking RBD antibodies; the latter’s induction of conformational changes in the S protein exposes 76E1’s epitope, thereby enhancing its membrane fusion inhibition [70]. A clear synergy exists between the FP antibody C77G12 and the ACE2-mimicking antibody S2E12, but not with S2M11, which locks the S protein in a closed state, indicating that synergy depends on epitope exposure and conformational transitions [73]. The cocktail composed of 1249A8 (targeting SH) and 1213H7 (targeting RBD) simultaneously blocks distinct key steps in viral entry, demonstrating significant synergistic neutralization against Omicron and superior in vivo efficacy [83]. However, CV3-25 (targeting SH) and CV3-1 (targeting RBD), despite fitting the “dual-blockade” concept, showed no obvious synergy, suggesting that cocktail potentiation depends on functional compatibility and potency balance between components [123].

Beyond neutralizing complementarity, cocktails can integrate Fc effector functions to enhance in vivo protection. For example, combining WRAIR-2125 (targeting RBD) with WRAIR-2039 (targeting NTD) improves in vivo protection [124] and CV3-13 combined with CV3-25 achieves 100% prophylactic protection [125]. The quadruple antibody combination TATX-03b’ not only neutralizes 17 SARS-CoV-2 variants but also significantly activates ADCC- and ADCP-related FcγR signaling in the combined state, demonstrating dual synergy in both neutralization and Fc-mediated functions [126]. Table 2 provides an overview of representative antibody cocktails against SARS-CoV-2, summarizing their components, target epitopes, and in vitro and in vivo synergistic effects.

## 5. Bi-/Multi-Specific Antibodies

**Figure 2:**
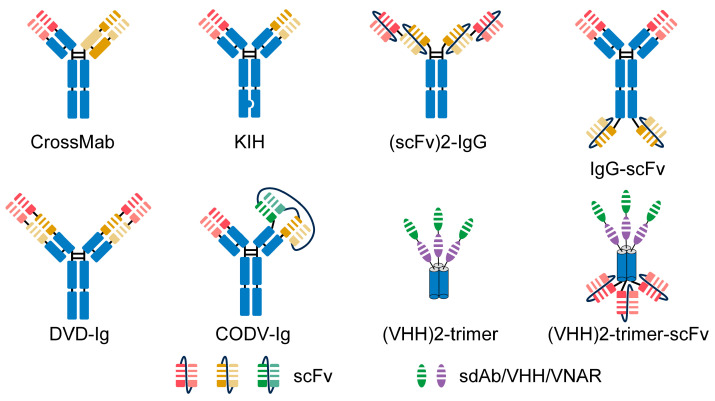
Schematic of representative bi-/multi-specific antibody formats. CrossMab, crossed monoclonal antibody; KIH, Knob-In-Hole; (scFv)2-IgG, dual single-chain Fv fused IgG; IgG-scFv, IgG with C-terminal scFv fusion; DVD-Ig, dual variable domain immunoglobulin; CODV-Ig, cross-over dual variable domains immunoglobulin; (VHH)2-trimer, dual VHH trimeric antibody; (VHH)2-trimer-scFv, dual VHH trimer with scFv fusion; scFv, single-chain variable fragment; sdAb, single domain antibody; VHH, variable domain of heavy chain of heavy-chain antibody; VNAR, shark variable new antigen receptor.

Bi-/multi-specific antibodies integrate multiple antigen-binding modules into a single molecule, enabling the coordinated combination of multiple antiviral mechanisms, including neutralization, conformational regulation, cross-linking/aggregation, and immune effector functions. Specifically, there are multiple integration formats, including Knobs-into-Holes, dual variable domain immunoglobulin (DVD-Ig), cross-over dual variable domain immunoglobulin (CODV-Ig), and single-chain variable fragment (scFv), among others (Figure 2). This integration can enhance neutralizing potency, breadth, and in vivo protective efficacy. Notably, weakly neutralizing or even non-neutralizing parental antibodies can be transformed into highly potent molecules through bi-/multi-specific engineering. For instance, bispecific or mutli-specific antibodies such as G9, Bis3, CoV-X4042, CoV-X2, COVA2-02+LY-CoV1404, 5-HI, Bis1-4, BA7208/7125, and Tri-1/Tri-2 all exhibit superior binding, neutralization, or in vivo protective activity compared to their parental counterparts [36,66,127,128,129,130,131,132,133]. Furthermore, the bispecific nanobody Bn03 and the trispecific antibody 7A9-19B8-S3_29 can further inhibit the virus by inducing S protein conformational changes or trimer dissociation [134,135].

Bi-/multi-specific designs can also effectively utilize parental antibodies whose epitopes overlap, making them difficult to combine in a traditional cocktail. Molecules such as Bi-Nab, bsAb1, Nb1-Nb2/Nb1-Nb2-Fc, and 3F-1B-2A demonstrate that through rational fusion design, competition between antibodies targeting overlapping epitopes can be alleviated, and occupancy and conformational restriction on the RBD or S1 can be enhanced, resulting in neutralization activity superior to that of the parental antibodies or their cocktail [136,137,138,139]. Among these, bsAb1 can sequentially occupy three “up”-conformation RBDs and restrict the dynamic changes of the S protein, highlighting the unique advantages of overlapping epitope antibodies within a multispecific framework [137].

The function of bi-/multi-specific antibodies is highly dependent on their molecular format and structural configuration. The activity can vary significantly when the same parental antibodies are used in different backbones. For example, among bispecific antibodies derived from B38/H4 and H11B11, the IgG-scFv format generally outperforms the DVD-Ig or CrossMab formats [140,141]. The bispecific antibody composed of B38/H4 has advanced to Phase Ia clinical studies [142]. Similarly, bispecific antibodies based on shark VNARs or camelid VHHs show that different heterodimeric or tandem formats involve trade-offs in affinity, neutralization, and ACE2-blocking capacity [143,144]. Beyond the backbone, the order of antigen-binding elements also determines functional output. For instance, in bispecific antibodies constructed from C1596/C952 and 7F3/GW01, the module order can significantly alter neutralizing activity by affecting RBD “up/down” transitions, subsequent antibody binding, and cross-linking capability [58,145]. Similar positional effects are also observed in Bi-Nab, KXD-BsAb02, and the trispecific CODV-Ig 61.1/46.1–182.1 [146,147,148].

In addition to inheriting the direct neutralization mechanisms of their parental antibodies, bi-/multi-specific antibodies often enhance potency through increased cross-linking and aggregation effects. Molecules such as CV1206_521_GS, FD01, BI-2C5B, and 61.1/46.1–182.1 can simultaneously bind multiple S proteins, forming cross-linked complexes or aggregates that significantly enhance viral inhibition [14,148,149,150]. Similarly, 14-H-06 exhibits stronger binding and neutralization than 14-crs-06 due to its ability to cross-link more S proteins [151]. Furthermore, bi-/multi-specific antibodies can be continuously optimized through multimerization or the introduction of additional functional modules. For example, the neutralizing activity of GS4 is further enhanced after trimerization [152]. The Fc-free trimer TNᵀ not only simultaneously blocks all six RBD binding sites on an S trimer but also leverages its DNGR-1 module to promote CD8^+^ T cell activation, demonstrating the unique potential of bi-/multi-specific platforms for integrated antiviral and immunomodulatory design [153]. Table 3 summarizes the representative bi-/multi-specific antibodies against SARS-CoV-2, including their molecular formats, parental antibodies, target epitopes, and enhanced characteristics relative to parental antibodies and cocktails.

## 6. Antibody Class Switching and Multivalent Modification

Mucosal immunity serves as the frontline barrier against viral invasion, in which secretory IgA (sIgA) holds unique advantages in preventing infection. Although plasma monomeric IgA typically exhibits lower neutralizing potency against SARS-CoV-2 compared to IgG, dimeric IgA shows an approximately 15-fold average increase in neutralizing activity against the same target [156]. This enhancement is primarily attributed to the ability conferred by the additional antigen-binding site to bridge adjacent S protein trimers and reduce dissociation rates [157]. Correspondingly, the dimeric IgA forms of antibodies such as Cv2.1169, MAb362, ZW2G10, H4, B38, and SA55 IgA1 all demonstrate superior neutralizing activity compared to their monomeric IgA and IgG counterparts [158,159,160,161,162]. Furthermore, the tetravalent secretory IgA bispecific antibody S2-3-IgA2m2, constructed based on aRBD2 and aSA3, exhibits stronger neutralization and better in vivo protection than its bispecific IgG form, and can effectively prevent Omicron BA.5 infection at low doses via intranasal administration [163].

IgG-based engineering represents another approach to enhance antibody efficacy. IgG3, due to its longer and more flexible hinge region, possesses higher bivalent binding efficiency. For example, the IgG3 form of REGN10933 shows over a 100-fold increase in neutralizing activity against the Beta and Omicron compared to its IgG1 form [164].

IgM-based engineering is another important strategy for antibody enhancement. Its advantages include significantly boosting neutralizing activity, reducing effective doses, and conferring additional inhibitory mechanisms such as virion aggregation. The NTD-targeting antibody N235, when engineered into the IgM format (MN235), shows markedly enhanced neutralization and improves intranasal prophylactic efficacy against Omicron BA.1 and XBB by inducing viral aggregation [105]. Similarly, IgM-14 can restore neutralization against IgG-14-resistant viruses and demonstrates superior in vivo protection at low doses [165]. The alpaca nanobody R14, after IgM conversion into MR14, also shows significantly better neutralization against Omicron BA.1, BA.2, and BA.4/5, as well as superior in vivo protection compared to the original molecule. Its mechanism extends beyond ACE2 blockade to include induction of virion aggregation [166].

Beyond engineering modifications mentioned above, multivalent modification is another effective route to enhance antibody potency. Trivalent nanobodies constructed via linkers or trimerization domains, such as Nb4-16t, 79C11-Trimer, and Nb6 Tribody, all exhibit stronger binding, neutralization, and in vivo protective capacity compared to their monovalent forms. The mechanisms primarily involve topological matching with the S protein trimer, simultaneous occupancy of multiple RBDs, and stabilization of specific conformations (e.g., the “3-RBD-down” state) [101,108,167]. Additionally, fusing nanobodies to an IgG Fc can enhance activity by increasing valency, as seen with 79C11, S2A9, W25, saRBD-1, 7F, and Nb12/Nb19 [100,108,109,168,169,170]. Further integration with nanoparticle platforms, such as conjugating 1C4, XMA01/04/09, or B-B2 to mi3 or AaLS particles via SpyTag/SpyCatcher, can further enhance binding stability and neutralizing activity. This is achieved by promoting S protein aggregation, virion cross-linking, increasing steric hindrance, and improving thermal stability, thereby amplifying the antiviral effect [171,172,173].

## 7. Fc Modification

The antiviral activity of antibodies in vivo is not solely dependent on neutralization; Fc-mediated effector functions can also contribute significantly to protection [174,175]. For example, the non-neutralizing NTD-targeting antibodies CV3-13 and DH1052 show markedly improved protective efficacy after the introduction of Fc-enhancing mutations [125,176]. Similarly, the S2-targeting non-neutralizing antibody COVA2-18, despite lacking neutralizing activity, exhibits prophylactic protection, and experiments have confirmed that this protection relies on intact Fc function [177].

For neutralizing antibodies, Fc function serves as an important complement to their in vivo efficacy. The LALA mutation in CV3-1 significantly impairs its prophylactic and therapeutic effects, while CV3-25 shows enhanced prophylactic activity after GASDALIE engineering [123]. The efficacy of the casirivimab/imdevimab cocktail and S309 also depends on FcR-mediated functions, with the strength of Fc effector activity significantly influencing therapeutic outcomes. However, the contribution of Fc function appears more limited in prophylactic settings, suggesting its role is influenced by the stage of infection and antibody titers [178].

Furthermore, IgG subclass and Fc engineering significantly impact the therapeutic performance of antibody drugs. IgG3, due to its stronger phagocytic and complement-activating capabilities, can further enhance Fc- and complement receptor-mediated phagocytosis when used in a cocktail format, possibly because it more readily forms the hexamers required for C1q activation [179]. However, the impact of Fc function on protection is not universally positive. For instance, the S2-targeting antibody 54043-5, which binds the conserved epitope aa 969–995, lacks neutralizing activity but can mediate ADCP. The wild-type antibody shows no in vivo protection, whereas the LALA-PG mutant version unexpectedly confers protection. This suggests that in some contexts, Fc effector functions might induce excessive inflammation, thereby offsetting their potential benefits [180].

Beyond directly modulating effector functions, Fc engineering can also indirectly enhance in vivo antibody potency by extending serum half-life. For example, both AZD8895 and AZD1061 incorporate the YTE modification to improve their persistence in vivo [181]. Table 4 summarizes representative engineered antibodies against SARS-CoV-2 that incorporate class switching, multivalent modification, or Fc engineering, highlighting the specific engineering strategies and their functional advantages.

## 8. Artificial Intelligence-Based Design and Optimization

With the advancement of AI, the potential of this emerging technology in the design and optimization of mAbs has gradually become apparent. In the context of COVID-19 antibody-related research and development, AI has also demonstrated unique advantages over conventional development approaches. In the field of de novo antibody design, the team at Vanderbilt University Medical Center proposed a sequence-based protein language model (PLM) termed MAGE (monoclonal antibody generator) for the de novo generation of SARS-CoV-2 RBD-specific antibodies. Wet-lab validation of 20 generated antibodies revealed that 45% achieved positive antigen binding, and some monoclonal antibodies exhibited KD values against the RBD at the nanomolar to sub-nanomolar level; among these, monoclonal antibodies RBD-409 and RBD-951 demonstrated broad-spectrum neutralizing potency spanning wild-type, Omicron BA.2, BJ.1, and BA.2.87.1 [185].

In the field of antibody optimization, Lawrence Livermore National Laboratory proposed a computational approach termed GUIDE (generative unconstrained intelligent drug engineering) that, under zero-shot conditions, achieved the restoration of COV2-2130’s neutralizing potency against Omicron within only three weeks. The best-performing antibody design, 2130-1-0114-112, carried only four amino acid substitutions, yet its IC_50_ values against Omicron BA.1 and BA.4 were improved by two orders of magnitude compared to the parental antibody [186]. Another study led by a team at Stanford University demonstrated, from an AI agent perspective, the potential of large language models (LLMs) in antibody optimization. This study employed GPT-4o to construct a virtual lab comprising an immunologist, a machine learning expert, a computational biologist, and a scientific critic. Through the introduction of point mutations, four existing nanobodies against the wild-type strain (Ty1, H11-D4, Nb21, and VHH-72) were optimized to enhance their binding affinity against Omicron KP.3. Some of the optimized antibodies showed improved binding to the wild-type and Omicron JN.1; however, few directly enhanced binding potency against the target Omicron KP.3 [187].

Overall, AI-based antibody design and optimization have yet to converge on a unified and effective research paradigm; nevertheless, multiple studies have provided preliminary evidence of their advantages over conventional wet-lab-based rational design approaches. With further iterative development in the future, AI-based antibody optimization and design may eventually achieve methodological maturity and serve as a viable replacement for traditional approaches.

## 9. Conclusions

The high mutability of SARS-CoV-2 has driven continuous iterative advancement in antibody technologies. Broadly neutralizing antibodies, nanobodies, antibody cocktails, bi-/multi-specific antibodies, and AI-driven antibody design and optimization have emerged as core innovation directions, each leveraging distinct advantages to overcome the limitations of conventional antibody approaches. These technologies collectively drive structural optimization and functional innovation in antibodies from multiple perspectives, and hold promise for addressing key challenges in anti-SARS-CoV-2 and pan-coronavirus antibody development, including insufficient neutralizing potency, low tolerance to escape mutations, and suboptimal in vivo protective efficacy. With the deep integration of genetic engineering, structural biology, and artificial intelligence, future antiviral antibody research and development will advance toward greater efficacy, broader spectrum coverage, and enhanced accessibility, thereby providing more robust technological support for global pandemic prevention and control, as well as preparedness against future “Disease X”.

## Figures and Tables

**Table 1:** Representative SARS-CoV-2-specific nanobodies.

Name	Origin	Target	Affinity	Neutralizing Activity Against Omicron Sublineages (IC_50_)	Neutralization Mechanism
Tnb04-1 [98]	Isolated from immunized alpacas	RBD, no competition with ACE2	WT RBD (K_D_ 0.648 nM)	BA.1, BA.2, BA.4/5, BA.5.2, BF.7, BQ.1, BQ.1.1, XBB, XBB.1, XBB.1.5, XBB.1.16, CH.1.1, EG.5, EG.5.1, BA.2.86, HK3, HV.1, JD.1.1, JN.1, KP.3, KP.3.1.1 (0.007–0.03 μg/mL)	Bound a conserved hydrophobic pocket in the RBD, disrupting formation of the proteinase K-resistant core required for viral-cell fusion.
Nb1 and Nb2 [99]	Phage library from immunized alpacas	RBD	Variants S1s (K_D_ 178–503 pM)	BA.1, BA.2, BA.3, BA.5, BA.2.75, BF.7, BQ.1, EG.5.1, XBB.1.5, JN.1 (2.82–799.3 ng/mL)	Directly interfered with RBD-ACE2 interaction.
Ma16B06, etc. [96]	Phage library from immunized alpacas	RBD	Variants RBDs (K_D_ 10–250 pM)	BA.1, BA.2.75, BA.5, BQ.1, BJ.1, XBB.1 (50–250 pM)	Occupied all three spike RBDs in both the up and down states.
W25 [100]	Isolated from immunized alpacas	RBD	Variants spikes (K_D_ 0.001–11.4 nM)	BA.1, BA.2 (1.45–2.07 nM)	Triggered premature fusogenic conformational changes in spike, leading to viral inactivation before cellular engagement.
Nb4 [101]	Synthetic nanobody phage library	RBD	BA.1 RBD (K_D_ 7.4 nM)	BA.1.1, BA.2 (1.5–20 μg/mL)	Competed with ACE2.
C5G2 [103]	Synthetic nanobody phage library	RBD	WT RBD (K_D_ 1.62 nM)	BA.1 (0.3 nM)	Steric hindrance from the FR2 of C5G2 inhibited ACE2-RBD binding.
3-2A2-4 [102]	Yeast library from immunized alpacas	RBD	NA	BA.1, BA.2, BA.2.12.1, BA.4/5 (0.032–0.17 μg/mL)	Suppressed the RBD transition from the down to the up state, thereby inhibiting ACE2 binding and viral entry.
1p1B10 [97]	Phage library from immunized Bactrian camel	RBM	XBB.1 RBD (K_D_ 0.73 nM)	BA.1, BA.2, BA.5, XBB.1, XBB.1.5, XBB.1.9.1, XBB.1.16, EG.5.1, JN.1, KS.1 (0.06–5.34 pM)	Competed with ACE2.
Nanosota-9 [95]	Phage library from immunized alpacas	RBD	BA.5, XBB.1.5, JN.1 spikes (K_D_ 0.06–29.4 nM)	BA.5, XBB.1.5, JN.1, BA.2.75, BQ.1, EG.5, KP.2 (1–15 ng/mL)	Two Nanosota-9 molecules crosslinked two spike RBDs.
IBT-CoV144 [104]	Isolated from immunized alpacas	RBD class 4	BA.2, BA.2.75, BA.4/5, BF.7, BQ.1.1 RBDs (22.3–52.9 nM)	BA.1, BA.2, BA.2.75, BA.4/5, BF.7, BQ.1.1 (331.4–722 ng/mL)	Crosslinked two spikes, inducing spike aggregation.
N235 [105]	Phage library from immunized alpacas	NTD	Variants spikes (K_D_ < 0.3 nM)	BA.1, BA.1.1, BA.2, BA.2.12.1, BA.2.3.20, BA.2.75, BA.3, BA.4/5, BQ.1.1, BF.7, XBB, XBB.1.5, XBB.1.16, CH.1.1, EG.5, EG.5.1, BA.2.86, JN.1 (0.3–7.2 μg/mL)	Interfered with neighboring RBDs and induced S1 shedding from the trimeric spike.
H17 and H145 [106]	Phage library from immunized alpacas	SH (1139–1152)	WT spike (K_D_ 11.3–17.9 nM)	BA.1, BA.2, BA.4/5, BA.2.12.1, BF.7, BA.2.75, XBB.1.5, XBB.1.16, JN.1, KP.3 (0.18–1.82 μg/mL)	Bound the unexposed inner face of SH, disrupting the S2 fusion machinery.
79C11 [108]	Phage library from immunized sharks	HR1 (916–935)	Variants spikes (K_D_ 20.3–35.0 nM)	BA.1, BA.2, BA.4, BF.7, BQ.1.1, XBB.1, XBB.1.5, XBB.1.16.1, EG.5, BA.2.86, JN.1, KP.2 (0.341–0.794 μg/mL)	Obstructed HR1-HR2 interaction and 6HB formation, thereby inhibiting virus–host membrane fusion.
S2A9 [109]	Phage library from immunized sharks	S2	WT S2 (K_D_ 590 nM)	BA.1, BA.2, BA.4/5 (2143–3733 ng/mL)	Interfered with S2-mediated viral-cell membrane fusion.
1p2B5 [110]	Phage library from immunized camel	NTD	XBB.1 NTD (K_D_ 0.44 nM)	XBB.1.9.1, EG.5.1, XBB.1.16, JN.1, KS.1 (0.17–1.23 nM)	Inhibited membrane fusion rather than blocking S-ACE2 binding.
R3DC23 [107]	Phage library from immunized llama	HR2	WT S-2P (K_D_ 0.17 nM)	BA.1, BA.2, BA.2.75, BA.5, BA.4.6, BQ.1.1, XBB, BA.2.86, EG.5.1, KP.3, KP.2.3, KP.1.1, LB.1, XEC (close to or below 1 ng/mL)	Clamped the prefusion HR2 coiled-coil and blocked 6HB formation.
BA.1-C2 and BA.1-D3 [111]	Phage library from immunized llama	HR2	BA.1 S2 (0.176 nM)	BA.1, EG.5.1, JN.1, XBB.1.5 (0.018–2.09 nM)	Clamped the prefusion HR2 coiled-coil and blocked 6HB formation.
B3 [112]	VL sdAb library from immunized rabbit	RBD, competition with ACE2	NA	BA.1 (5.3 μM)	Competed with ACE2.

NA, not available.

**Table 2:** Representative of antibody cocktails against SARS-CoV-2.

Name	Type	Components	Epitopes	Neutralizing Activity Against Omicron Sublineages (IC_50_)	In Vitro Synergistic Effects (Binding, Neutralization, ADCC)	In Vivo Synergistic Effects (Animal Models)
9E12+10D4+2G1 [120]	3-antibodies cocktail	9E12	RBD group 1	BA.1 (4258 ng/mL)	The cocktail remained resistant to viral escape after 30 passages and exhibited mutation tolerance far greater than either antibody alone.	NA
		10D4	RBD group 2			
		2G1	RBD group 3			
7B9-9D11+2G1 [120]	bispecific + antibody cocktail	7B9-9D11	RBD group 2/1	BA.1 (1138 ng/mL)	Exhibited complementary neutralization spectra.	NA
		2G1	RBD			
XMA01+XMA04+XMA09 [48]	3-antibodies cocktail	XMA01	RBD class 1	BA.1 (15.6 ng/mL)	Exhibited synergistically enhanced neutralizing activity against Omicron.	The XMA01/XMA04 dual-antibody cocktail exhibited synergistically enhanced in vivo protective efficacy (Syrian golden hamsters).
		XMA04	RBD class 2/3			
		XMA09	RBD class 5			
IMM-BCP-01 [114]	3-antibodies cocktail	IMM20184	RBD	BA.1 (62.89 nM)	Exhibited synergistically enhanced neutralizing activity against early VOCs and Omicron BA.1/BA.2, and synergistically enhanced ADCC, ADCP, and CP.	Exhibited synergistically enhanced in vivo prophylactic and therapeutic efficacy against Omicron BA.1 (Syrian golden hamsters).
		IMM20190	RBD			
		IMM20253	RBD			
B1-182.1+A19-46.1 [8]	2-antibodies cocktail	B1-182.1	RBD class 1	BA.1 (28.3 ng/mL)	Exhibited synergistically enhanced neutralizing activity against Omicron.	-
		A19-46.1	RBD class 2			
H014+HB27+P17+FC05 [117]	3/4-antibodies cocktail	H014	RBD	NA	Exhibited synergistically enhanced spike binding.	NA
		HB27 alternate	RBD			
		P17	RBD			
		FC05	NTD			
WRAIR-2125+WRAIR-2039 [124]	2-antibodies cocktail	WRAIR-2125	RBD class 1	NA	Exhibited complementary neutralization spectra and increased the genetic barrier to viral escape.	Showed significant in vivo prophylactic and therapeutic synergy (K18-hACE2 transgenic mice).
		WRAIR-2039	NTD antigenic supersite			
EGH [115]	3-antibodies cocktail	13H7	NTD	BA.1, BA.2, BA.2.12.1, BA.5 (270–1091 ng/mL)	Exhibited complementary neutralization spectra.	NA
		9G11	RBD			
		3E2	RBD			
76E1+(ACE2/CB6/P2C-1F11/28-8L) [70]	2-antibodies cocktail	76E1	FP	NA	Exhibited synergistically enhanced neutralizing activity against SARS-CoV-2.	NA
		ACE2/CB6/P2C-1F11/28-8L	RBD class 1			
S2E12+C77G12 [73]	2-antibodies cocktail	C77G12/VN01H1	FP	NA	Exhibited synergistically enhanced neutralizing activity against SARS-CoV-2.	NA
		S2E12	RBD			
1249A8+1213H7 [83]	2-antibodies cocktail	1249A8	SH	BA.1 (25.8–1338 ng/mL)	Exhibited synergistically enhanced neutralizing activity against Omicron.	Showed in vivo prophylactic synergy in mice against Beta and Omicron, and therapeutic synergy in hamsters against Delta and SARS-CoV (K18 hACE2 transgenic mice, syrian golden hamster).
		1213H7	RBD			
CV3-1+CV3-25 [123]	2-antibodies cocktail	CV3-1	RBD	NA	Did not exhibit synergistic enhancement of in vitro neutralizing activity or Fc effector functions.	Did not exhibit synergistic enhancement in the prophylactic regimen (K18 hACE2 transgenic mice).
		CV3-25	SH			
TATX-03b′ [126]	4-antibodies cocktail	23-H7	RBD	BA.1, BA.1.1, BA.2, BA.4/5, BA.2.12.1, BA.2.75 (20–8020 ng/mL)	Exhibited synergistically enhanced neutralizing activity against Omicron BA.4/BA.5, and synergistically enhanced in vitro ADCC and ADCP.	Exhibited synergistically enhanced in vivo viral clearance and alleviated lung pathology (Syrian golden hamster).
		21-F2(N92Q)	RBD			
		22-F7	CT1			
		2-A6	S2			
SA55+SA58 [18]	2-antibodies cocktail	SA55	RBD	BA.1, BA.1.1, BA.2, BA.3, BA.2.13, BA.2.12.1, BA.4/5 (3.2–7.8 ng/mL)	Exhibited synergistically enhanced neutralizing activity against Omicron.	NA
		SA58	RBD			

NA, not available.

**Table 3:** Representative of bi-/multi-specific antibodies against SARS-CoV-2.

Name	Format	Parent Antibodies	Epitope	Neutralizing Activity Against Omicron Sublineages (IC_50_)	Enhanced Characteristics
FD01 [150]	(scFv)2-IgG	GW01	RBD class 4	BA.1, BA.1.1, BA.2 (69.6–1285 ng/mL)	Exhibited significantly greater binding activity, neutralizing activity, and mutation tolerance than parental antibodies and cocktails.
		16L9	RBM		
G9 [127]	(scFv)2-IgG	GW01	RBM	BA.1 (1257 ng/mL)	Exhibited significantly greater neutralizing activity than parental antibodies.
		REGN10989	RBD, ACE2 non-competing		
G7-Fc [145,154]	(scFv)2-IgG	GW01	RBD, ACE2 non-competing	BA.1, BA.2, BA.2.12.1, BA.2.75, BL.1, BA.2.75.2, BA.2.75.4, BN.2.1, CA.1, BA.2.87.1, BA.2.86, BR.1, CH.1.1, XBB, XBB.1.5, XBB.1.16, XBB.1.16.1, BA.5, BR.1, BF.7, BA.4.6, BA.5.1.12, BA.5.6.2, BU.1, BQ.1.1, EG.5, JN.1, KP.2 (0.0191–4.3 nM)	Exhibited significantly greater neutralizing activity and in vivo prophylactic and therapeutic efficacy than parental antibodies and cocktails.
		7F3	RBD class 2		
Bi-Nab [136]	(scFv)2-IgG	35B5	RBD class 3	BA.1, BA.2 (31.6–399.2 ng/mL)	Bi-Nabs exhibited stronger binding and neutralizing activity against early VOCs than parental antibodies and cocktails, and markedly greater neutralizing activity against Omicron BA.1 and BA.2.
		32C7	RBD class 3		
Bi-Nab [146]	(scFv)2-IgG	35B5	RBD class 3	BA.1, BA.2 (0.15–0.67 nM)	Exhibited greater RBD binding but lower S2 binding than parental antibodies, while showing broader neutralization breadth and greater neutralizing potency than parental antibodies and cocktails.
		47D10	S2		
bn03 [134]	tandem link (sdAb)2	n3130v	RBD, ACE2 non-competing	BA.1 (100 ng/mL, Inferred from the neutralization curve)	Exhibited significantly greater neutralizing activity than parental antibodies and cocktails.
		n3113v	RBD, ACE2 non-competing		
bsAb15 [140]	IgG-scFv	B38	RBD (around K417, N460)	NA	Exhibited significantly greater neutralizing activity, mutation tolerance, and in vivo prophylactic and therapeutic efficacy than parental antibodies and cocktails.
		H4	RBD (around E484)		
CoV-X2 [129]	CrossMAb	C121	RBD (around E484)	NA	Exhibited complementary neutralization spectra and significantly greater neutralizing activity than parental antibodies.
		C135	RBD (around R346)		
14-H-06 [151]	IgG-scFv	CoV2-14	RBD class 2	BA.1 (1110 ng/mL)	14-H-06 exhibited significantly greater binding activity, neutralizing potency, neutralization breadth, and in vivo therapeutic efficacy than parental antibody cocktails and 14crs-06.
		CoV2-06	RBD class 1		
Bis4 [132]	VH/Fab IgG KIH	VH B01	RBD B site	NA	Exhibited significantly greater neutralizing potency than parental antibodies and cocktails.
		Fab D01	RBD D site		
CV1206_521_GS [149]	DVD-Ig	CV1206	RBD, ACE2 competing	NA	Exhibited significantly greater neutralizing activity than parental antibodies and cocktails.
		CV521	NTD supersite		
CoV2-biRN5 [58]	(scFv)2-IgG	C1596	NTD not supersite	BA.1, BA.2, BA.4/5, XBB.1.5, XBB.1.16, EG.5.1, HV.1, BA.2.86, JN.1 (1–5 ng/mL)	CoV2-biRN5 exhibited markedly greater neutralizing activity against the tested viral strains than parental antibodies, cocktails, CrossMab, and DVD-Ig-format bispecific antibodies.
		C952	RBD class 3		
KXD-BsAb02 [147]	IgG-scFv	KXD643	RBD-8	BA.1, BA.4/5, BA.2.75, BQ.1, XBB.1.5, EG.5.1, HK.3, BA.2.86, JN.1, KP.2, KP.3 (30–145 ng/mL)	Exhibited significantly greater neutralizing activity than parental antibodies and cocktails.
		KXD686	NTD site iv		
bsAb1 [137]	IgG-scFv	H4B6	RBD	BA.1, BA.2, BA.2.75, BA.2.76, BA.4/5, BA.4.6, BF.7, XBB.1.5, XBB.1.16, XBB.2.3, BA.2.86, JN.1 (2–17 ng/mL)	Exhibited significantly greater binding activity, neutralization breadth, and neutralizing potency than parental antibodies and cocktails.
		H4D12	RBD		
61.1/46.1–182.1 [148]	CODV-Ig	A19-61.1	RBD class 3	BA.1.1, BA.2, BA.2.12.1, BA.4/5 (26.2–1053 pM)	Exhibited significantly greater neutralizing activity than parental antibodies and significantly greater mutation tolerance than parental antibodies and cocktails.
		A19-46.1	RBD class 2		
		B1-182.1	RBD class 1		
H11B11_m336 [141]	IgG-scFv	H11B11	ACE2	BA.2, BA.5, XBB.1.5 (251–424 ng/mL)	Simultaneously neutralized SARS-CoV, SARS-CoV-2, and MERS-CoV, and exhibited significantly greater neutralizing activity than parental antibodies and cocktails.
		m336	MERS-CoV S		
Bis3 [128]	IgG-scFv	CvMab-6	RBD	BA.1 (13.1 nM)	Exhibited significantly greater neutralizing activity than parental antibodies and cocktails.
		CvMab-62	S2 (1123–1148)		
COVA2-02+LY-CoV1404 [130]	cFAE	COVA2-02	RBD unique epitope	BA.4/5, BQ.1.1, XBB.1 (2–13,600 ng/mL)	Exhibited greater neutralizing activity than parental antibodies against some viruses, but variable performance relative to cocktails.
		LY-CoV1404	RBD class 3		
BA7208+BA7125 [36]	scFv/Fab IgG KIH	BA7208	RBD class 3	BA.1, BA.2, BA.2.12.1, BA.2.13, BA.3, BA.4/5 (31.98–92.81 ng/mL)	Exhibited complementary neutralizing activities and synergistically enhanced binding to the RBDs of Mu and Omicron BA.1.
		BA7125	RBD class 1		
BI-2C5B [14]	IgG-scFv	6-2C	RBD class 3	BA.1, BA.2, BA.2.12.1, BA.2.75, BA.2.75.2, BN.1, BA.3, BA.4, BA.4.6, BA.5, BF.7, BQ.1, BQ.1.1, XD, XBB, XBB.1.5 (6–97 ng/mL)	Exhibited neutralizing activity comparable to or greater than that of parental antibodies, effectively neutralized variants partially resistant to parental antibodies, and provided superior in vivo prophylactic protection at lower doses than parental antibodies and cocktails.
		10-5B	RBD class 1		
5-HI [131]	DVD-Ig	SCM 13–65	S1	BA.1, BA.2, BA.3, BA.5, BA.2.75, BQ.1, BF.7, XBB.1.5, EG.5.1, JN.1 (0.008–12.74 nM)	Exhibited significantly greater binding activity, neutralization breadth, and neutralizing potency than parental antibodies and cocktails.
		SCM 15–45	S1		
CoV-X4042 [66]	CrossMab	sd1.040	SD1	BA.1, BA.2, BA.2.75, BA.2.75.2, BA.4/5 (1000 ng/mL, inferred from the neutralization curve)	Exhibited greater neutralizing activity than parental antibodies and cocktails.
		rbd.042	RBD		
ShAb01H02K [143]	VNAR-IgG KIH	ShAb01	RBD class 4	BA.1, BA.5 (391–524 ng/mL)	Exhibited significantly greater binding activity, neutralization breadth, and neutralizing potency than parental antibodies.
		ShAb02	RBD class 3		
7A9-19B8-S3_29 [135]	tandem link (VHH)3	7A9	RBD 353–364	NA	Exhibited significantly greater neutralizing activity than parental antibodies and cocktails.
		19B8	NTD		
		S3_29	apex of S2		
TNᵀDNGR-1 [153]	(VHH)2-trimer-scFv	E	RBD	NA	TNᵀ and TNᵀDNGR-1 exhibited significantly greater neutralizing activity against Omicron BA.1 than TN; whereas parental antibodies (TN and TNᵀ) failed to protect against lethal infection, in vivo protection improved from ineffective to complete.
		V	RBD		
		7H11	DNGR-1		
Tripod-GS4r [152]	sybody1-sybody2-trimer	Sb#15	RBD, ACE2 competing	NA	Exhibited significantly greater neutralizing activity than parental antibodies and cocktails.
		Sb#68	RBD, ACE2 non-competing		
NB1A7+NB1B11 [144]	(VHH)2-IgG, etc.	NB1A7	RBD, ACE2 non-competing	NA	Exhibited significantly greater neutralizing activity than parental antibodies.
		NB1B11	RBD, ACE2 competing		
Nb1-Nb2-Fc [138]	(VHH)2-IgG	Nb1	RBD, ACE2 non-competing	BA.1, (0.0017 nM)	Exhibited significantly greater neutralizing activity than parental antibodies and cocktails.
		Nb2	RBD, ACE2 competing		
3F-1B-2A [139]	(VHH)3-IgG	3F	RBD, ACE2 non-competing	NA	Exhibited significantly greater binding activity and neutralizing activity than parental antibodies and cocktails.
		1B	RBD, ACE2 competing		
		2A	RBD, ACE2 competing		
Tri-1 [133]	KIH + CrossMab + IgG-scFv	PW5-570	RBM	BA.1, XBB.1, EG.5.1, JN.1, JN.1.7, KP.2, KP.3.1.1 (0.034–19.08 nM)	Tri-1/Tri-2 exhibited higher binding affinity, greater neutralizing potency, broader sarbecovirus coverage, and lower viral escape risk than parental mAbs and cocktails.
		PW5-5	RBD		
		PW5-535	RBD nearby SD1		
N-14-44-scFv [155]	scFv-Fab-IgG	P14-44	RBD, noRBM	BA.1 (2.02 ng/mL)	N-14-44-scFv exhibits the highest neutralization potency, broadest activity against Omicron sublineages and superior in vivo protection compared with other bsAb formats, parental antibodies, and the cocktail.
		P5-22	RBM		

NA, not available.

**Table 4:** Representative of other engineered antibodies against SARS-CoV-2.

Name	Original Antibody Type	Target	Format	Engineering Advantages	Neutralizing Activity Against Omicron Sublineages (IC_50_)
W25-Fc [100]	VHH from alpacas	RBD	human IgG Fc	W25-Fc exhibited greater neutralizing activity than W25, particularly by simultaneously engaging two up-state RBDs.	BA.1, BA.2 (1.45–2.07 nM)
S2A9-hFc [109]	VNAR from sharks	S2	human IgG1 Fc	S2A9-hFc exhibited greater neutralizing activity than its monomeric form due to bivalent effects.	BA.1, BA.2, BA.4/5 (976–1854 nM)
MR14 [166]	VHH from alpacas	RBD	huIgG1, trivalent tandem nanobody, human IgM	These variants exhibited higher RBD affinity, greater neutralizing activity against Omicron sublineages, and improved in vivo protective efficacy; they also induced spike aggregation.	BA.1, BA.2, BA.2.12.1, BA.2.75, BA.3, BA.4/5 (0.16–18.7 ng/mL)
79C11-Fc/79C11-Dimer/79C11-Trimer [108]	VNAR from sharks	HR1 (916–935)	bivalent huIgG1 Fc/bivalent/trivalent tandem nanobody	Multivalent effects significantly enhanced binding affinity and neutralizing activity.	BA.1, XBB.1.5, BA.2.86, JN.1 (0.011–0.075 μg/mL)
Bi-saRBD-1/Fc-saRBD-1 [168]	VHH from alpacas	RBD class 1	(VHH)2, human IgG Fc	Exhibited greater RBD affinity due to multivalent effects.	NA
7F-7F/7F-Fc [169]	VHH from llamas	RBD class 4	(VHH)2, human IgG Fc	Exhibited greater neutralizing potency and broader neutralization breadth due to multivalent effects.	BA.2, BA.5 (0.31–0.95 μM)
Nb19 trimer, etc. [170]	VHH from alpacas/nanomice	RBD	(VHH)3-human IgG1 Fc	Multimers exhibited greater binding avidity and neutralizing potency, compensating for mutation-induced affinity loss, and simultaneously cross-linked multiple spikes.	NA
Nb4-16t [101]	Synthetic nanobody	RBD	trivalent tandem nanobody	Exhibited greater RBD affinity, enhanced neutralizing activity, broader neutralization breadth, and improved in vivo protective efficacy.	BA.1.1, BA.2 (0.4–1.9 μg/mL)
DXP-604 mIgA1/dIgA1/sIgA1, etc. [157]	human IgG	RBD class 1	monomeric/dimeric/secretory IgA1	The longer hinge of IgA1 conferred greater flexibility, and the multivalent effect of dimeric IgA significantly enhanced neutralizing activity.	BA.1, BA.2, BA.5 (0.03–3.43 nM)
H4-IgA1-m, H4-IgA1-d, etc. [161]	human IgG1	spike	monomeric/dimeric IgA1	The long hinge region and multivalency of dimeric IgA enhanced viral spike cross-linking.	NA
ZW2G10 (sIgA1) [160]	human IgG1	RBD, no competition with ACE2	human IgG1/mIgA1/dIgA1/sIgA1/mIgA2/dIgA2/sIgA2	The IgA form exhibited greater binding affinity, enhanced neutralizing activity, and improved in vivo protective efficacy due to its longer flexible hinge, multivalent binding, and mucosal adaptation.	BA.2.75, BA.2.76, BA.4/5 (3.513–61.97 ng/mL)
REGN10933 IgG3 [164]	human IgG1	RBD class 1	human IgG3	The IgG3 form exhibited greater binding avidity and neutralizing potency owing to its long flexible hinge.	BA.1 (0.34 nM)
IgM-14 [165]	human IgG	RBD	human IgM	IgM-14 exhibited greater neutralizing potency, broader neutralization breadth, and improved in vivo protective efficacy due to multivalent effects and mucosal adaptation.	NA
MN235 [105]	VHH from alpacas	NTD	human IgM	MN235 exhibited enhanced neutralizing activity and superior in vivo protective efficacy owing to its ability to cross-link virions.	BA.1, BA.1.1, BA.2, BA.4, BA.5, BF.7, XBB, EG.5.1 (0.012–0.941 μg/mL)
Nb6 tribody [167]	VHH from camelid	RBD	tribody	Tribody exhibited greater binding avidity and neutralizing potency due to multivalent effects.	NA
mi3-1C4 [171]	Mouse IgG	RBD class 3	mi3 nanoparticle by SpyTag/SpyCatcher	Exhibited greater binding affinity, enhanced neutralizing activity, broader neutralization breadth, and improved in vivo protective efficacy due to multivalent effects that promoted spike aggregation.	BA.1, BA.1.1, BA.2, BA.2.12.1, BA.2.3.20, BA.2.75, BA.3, BA.4/5, BQ.1.1, BF.7, XBB, XBB.1.5, XBB.1.16, CH.1.1, EG.5, EG.5.1, BA.2.86, JN.1 (0.003–1.85 μg/mL)
XMA04-mi3 [172]	Mouse IgG	RBD class 2/3	mi3 nanoparticle by SpyTag/SpyCatcher	Exhibited greater binding affinity, enhanced neutralizing activity, broader neutralization breadth, and enhanced ADCC due to multivalent effects that promoted spike aggregation.	BA.1, BA.1.1, BA.2, BA.2.12.1, BA.2.75, BA.4/5, XBB (2.6–6.3 ng/mL)
LS-B-B2 [173]	VHH from alpacas	RBD	AsLS nanoparticle by SpyTag/SpyCatcher	Exhibited enhanced neutralizing activity and improved thermal stability due to multivalent effects and nanoparticulation.	BA.1 (0.653 μg/mL)
SA55-IgA [162]	Human IgG	RBD	Human mono/dimer/secretory-IgA1	IgA formats markedly enhanced or restored neutralization against IgG-resistant mutants.	BA.1, BA.2, BA.4/5, BA.2.75, BF.7, CH.1.1, BQ.1.1, XBB.1, XBB.1.5, XBB.1.16, XBB.1.16.1, EG.5.1, BA.2.86, JN.1, JN.1 FLiRT, BA.2.87.1 (0.0009–0.1900 nM)
IgM-14 [182]	Human IgG	RBD	Human IgM	IgM-14 exhibited greater neutralizing potency, broader neutralization breadth, and a higher resistance barrier than parental IgG-14.	BA.1, BA.2, BA.3 (0.54–23.9 μg/mL)
2D4-IgA [183]	VHH from alpacas	RBD	Human IgA1	2D4-IgA exhibited greater and broader neutralizing activity, extended half-life, and effective mucosal delivery.	BA.1, BA.4, BA.5, XBB (21.3–313.1 ng/mL)
Nb4×3 [184]	VHH from dromedaries	RBD	Tandem trimer	Nb4×3 exhibited greater neutralizing potency and broader activity against SARS-CoV-2 variants, especially escape sublineages.	BA.1, BA.5, BQ.1.1 (0.05–4.32 nM)

NA, not available.

## Data Availability

No new data were created.

## Abbreviations

The following abbreviations are used in this manuscript:

mAb	Monoclonal antibody
nAb	Neutralizing antibody
RBD	Receptor-binding domain
NTD	N-terminal domain
SD1	Subdomain 1
FP	Fusion peptide
SH	Stem helix
TMPRSS2	Transmembrane serine protease 2
AI	Artificial intelligence

## Appendix A

**Table A1:** Representative SARS-CoV-2 broadly neutralizing antibodies and their neutralizing activity against Omicron sublineages.

Name	Target	Neutralizing Activity Against Omicron Sublineages (IC50)
BA7535 [11]	RBD class 1	BA.1, BA.1.1, BA.2, BA.2.12.1, BA.2.13, BA.2.38.1, BA.2.74, BA.2.75, BA.2.76, BA.2.77, BA.2.79, BA.2.80, BA.3, BA.4/5, BA.4.6, BA.4.7, BA.5.5.1, BF.7, BQ.1, BQ.1.1, XBB.1, XBB.1.5, XBB.1.9.1, CH.1.1, EG.5 (0.3789–274.4 ng/mL)
17T2 [12]	RBD class 1	BA.1, BA.2, BA.4/5, BQ.1.1, XBB.1.5, XBB.1.16, EG.5.1, BA.2.86 (3.2–1180 ng/mL)
87G7 [13]	RBD class 1	BA.1, BA.2 (6.7–10.2 ng/mL)
10-5B [14]	RBD class 1	BA.1, BA.2, BA.2.12.1, BA.2.75, BN.1, BA.3, XD (4–16 ng/mL)
AZD3152 [15]	RBD class 1	BA.1, BA.1.1, BA.2, BA.2.12.1, BA.4/5, BA.2.75, BA.4.6, BA.4.7, BA.5.9, BA.2.75.2, BF.7, BQ.1, BQ.1.1, XBB, XBB.1, XBB.1.5, XBB.1.16, BA.2.86, JN.1, XBB.1.5.10, EG.5.1, HV.1, HK.3 (3.2–2286.2 ng/mL)
S2K146 [17,18,19,20]	RBD class 1	BA.1, BA.1.1, BA.2, BA.3, BA.2.13, BA.2.12.1, BA.4/5, XBB.1.5, EG.5.1, HK.3 (11–4147 ng/mL)
GAR05 [21]	RBD class 1	BA.1, BA.2, BA.5 (71.31–337.6 ng/mL)
ZCP3B4 [22]	RBD class 1	BA.1, BA.2, BA.2.12.1, BA.2.75, BA.4/5, BF.7, BE.1.1, BQ.1.1, XBB, XBB.1, XBB.1.5, HK.3, HV.1, EG.5.1, BA.2.86, JN.1, KP.2 (2.7–9.8 ng/mL)
P2-1B1 [23]	RBD class 1	BA.1, BA.2, BA.2.12.1, BA.2.75, BA.3, BA.4/5 (7.6–1619.2 ng/mL)
P4J15 [24]	RBD class 1	BA.1, BA.4/5, BA.2.75.2, BQ.1, BQ.1.1, XBB.1, XBB.1.5, CH.1.1, XBB.1.16, XBB.1.16.1, XBB.2.3, EG.1, EG.5.1 (2–18 ng/mL)
KXD03 [25]	RBD class 1	BA.1, BA.2, BA.3, BA.4/5, BA.2.75, BF.7, BQ.1, XBB, XBB.1, XBB.1.5, XBB.1.16, EG.5, EG.5.1, FL.1.5, FL.1.5.1, HK.3 (22–489 ng/mL)
VIR-7229 [26]	RBD class 1	BA.1, BA.2, BA.2.3, BA.2.75.2, BA.5, BQ.1.1, XBB.1.5, CH.1.1, XBB.2.3, EG.5, EG.5.1, XBB.1.16, XBB.1.16.1, XBB.1.16.6, FL.1.5.1, HV.1, XBB.1.5.70, HK.3, JD.1.1, BA.2.86, JN.1, JN.1.16, JN.1.18, BA.2.87.1 (1.34–434.5 ng/mL)
BD55-1205 [27]	RBD class 1	BA.1, BA.2, BA.5, BQ.1.1, XBB.1.5, HK.3.1, JN.1, KP.3 (3–12 ng/mL)
P5-1C8 [23,28]	RBD class 1	BA.1, BA.2, BA.2.12.1, BA.2.75, BA.3, BA.4/5, JN.1 (7.7–21 ng/mL)
P5S-2A9 [23]	RBD class 2	BA.1, BA.2, BA.2.12.1, BA.2.75, BA.3, BA.4/5 (13.4–1102.4 ng/mL)
D1F6 [29]	RBD class 2	BA.1, BA.1.1, BA.2, BA.2.12.1, BA.2.13, BA.3, BA.4/5, BQ.1.1, XBB.1.5 (15–5038 ng/mL)
A19-46.1 [8]	RBD class 2	BA.1 (223 ng/mL)
COV2-2196 [8,30]	RBD class 2	BA.1, BA.1.1, BA.2, BA.2.12.1 (361–704 ng/mL)
S309 [20,32]	RBD class 3	BA.1, BA.1.1, BA.2, BA.2.12.1, BA.2.75, BA.4/5, BF.7, BQ.1, BQ.1.1, XBB, XBB.1.5, EG.5.1, HK.3, BA.2.86, JN.1 (252.7–11,742 ng/mL)
SP1-77 [33]	RBD class 3	BA.1, BA.2, BA.3, BA.4/5, BA.2.12.1 (6.5–33 ng/mL)
SW186 [34]	RBD class 3	BA.1 (332 ng/mL)
LY-CoV1404 [20,35,130]	RBD class 3	BA.1, BA.2, BA.4/5, BQ.1.1 (0.4–27 ng/mL)
BA7208 [36]	RBD class 3	BA.1, BA.2, BA.2.12.1, BA.2.13, BA.3, BA.4/5 (1.24–5.52 ng/mL)
S2X324 [37]	RBD class 3	BA.1, BA.2, BA.4-V3G, BA.5, BA.2.12.1 (49.8–2650.7 ng/mL)
P2S-2E9 [23]	RBD class 3	BA.1, BA.2, BA.2.12.1, BA.2.75, BA.3, BA.4/5 (8.1–3129.2 ng/mL)
6-2C [14]	RBD class 3	BA.1, BA.2, BA.2.12.1, BA.2.75, BA.2.75.2, BN.1, BA.3, BA.4, BA.4.6, BA.5, BF.7, BQ.1, BQ.1.1, XD, XBB, XBB.1.5 (194–3584 ng/mL)
1G1 [32]	RBD class 3	BA.1, BA.1.1, BA.2, BA.2.12.1, BA.2.75, BA.4/5 (1.2–19.6 ng/mL)
ADG20 [14,30,42]	RBD class 4	BA.1, BA.1.1, XD (181–1203 ng/mL)
DH1047 [30,44]	RBD class 4	BA.1, BA.1.1 (4322–5723 ng/mL)
AB2-122 [46]	RBD class 4	BA.1, BA.2, BA.3, BA.5, BQ.1.1, XBB.1.5, BA.2.86, JN.1, KP.2, KP.3, KP.3.1.1, LP.8.1.1, MC.10.1, NB.1.8.1, XFG (20–1170 ng/mL)
S2H97 [20,47,49]	RBD others	BA.1, BA.2, BA.5, BQ.1.1, XBB.1.5, EG.5.1, HK.3, BA.2.86, JN.1 (224–1539 ng/mL)
C68.61 [49,67]	RBD others	BA.1, BA.2, BA.2.12.1, XBB, XBB.4, XBB.1.5, BA.4/5, BQ.1.1, EG.5.1, JN.1 (120–850 ng/mL)
GAR12 [21]	RBD others	BA.1, BA.2, BA.5 (16.17–62.59 ng/mL)
ION_300 [20,50]	RBD others	BA.2, XBB.1.5, EG.5.1, HK.3, BA.2.86, JN.1 (343–3838 ng/mL)
5–7 [54]	NTD supersite	BA.1, BA.1.1, BA.3 (100–1000 ng/mL, Inferred from the neutralization curve)
C1717 [55]	NTD supersite	BA.1 (507 ng/mL)
C1520 [55]	NTD supersite	BA.1 (31 ng/mL)
C1791 [55]	NTD supersite	BA.1 (167 ng/mL)
BD58-0706 [56]	NTD N1/N2 loops	BA.2, BA.2.75, BA.5, BQ.1.1, XBB, XBB.1, XBB.1.5, EG.5.1, HK.3.1 (1–35 ng/mL)
K501SP6 [57]	NTD close to SD1	BA.1, BA.2, BA.5, BQ.1.1.20, XBB.1, BA.2.86, JN.1 (3.06–25.9 μg/mL)
C1596 [58]	quaternary epitope containing the NTD, RBD and SD1	BA.1, BA.4/5, XBB.1.5, EG.5.1, BA.2.86, JN.1 (57–4450 ng/mL)
S3H3 [59,60,61,62,63]	SD1	BA.1, BA.2, XBB.1, XBB.1.5, EG.5.1, HK.3 (24–41 ng/mL)
SD1-1 [64]	SD1	BA.1, BA.1.1, BA.2, BA.2.12.1, BA.2.75, BA.4/5, BA.4.6, BA.2.75.2, BA.2.3.20, BA.2.10.4, BQ.1, BQ.1.1, BS.1, BF.7, BJ.1, BN.1, CH.1.1, CA.3.1, XBB, XBB.1, XBB.1.5, XBB.1.5.10, XBB.1.5.70 (12–66 ng/mL)
P008_60 [65]	SD1	BA.1 (10–100 μg/mL, Inferred from the neutralization curve)
Sd1.040 [66]	SD1	BA.1, BA.2, BA.2.75, BA.2.75.2, BA.4/5 (1004–4486 ng/mL)
C68.59 [67]	SD1	BA.1, BA.2, BA.2.12.1, XBB, XBB.4, XBB.1.5, BA.4/5, BQ.1.1 (34–156 ng/mL)
MO11 [68]	SD1	BA.1, BA.2, BA.2.75, BA.5, BQ.1.1, XBB.1, XBB.1.5, XBB.1.16, EG.5.1 (110.3–467.6 ng/mL)
76E1 [70]	FP	BA.1 (100 ng/mL, Inferred from the neutralization curve)
C20.119 [71]	FP	BA.1, BA.2, BA.5, BQ.1.1, XBB, XBB.1.5 (19–59 μg/mL)
COV44-62 [72]	FP	BA.1, BA.2, BA.4/5 (10.38–51.89 μg/mL)
COV44-79 [72]	FP	BA.1, BA.2, BA.4/5 (33.02–55.44 μg/mL)
VN01H1 [73]	FP	BA.1, BA.2 (12.5–19.7 μg/mL)
VP12E7 [73]	FP	NA
C77G12 [73]	FP	BA.1, BA.2 (2.9–5.4 μg/mL)
fp.007 [66]	FP	BA.1, BA.2, BA.2.75, BA.2.75.2, BA.4/5 (1889–14,469 ng/mL)
S2P6 [66,74]	SH	BA.1, BA.2, BA.2.75, BA.2.75.2, BA.4/5 (29,351–68,964 ng/mL)
CV3-25 [66,75,76]	SH	BA.1, BA.2, BA.2.75, BA.2.75.2, BA.4/5 (571.9–2469 ng/mL)
CC40.8 [79,85]	SH	NA
WS6 [81]	SH	BA.1 (3.43 μg/mL)
1249A8 [83]	SH	NA
hr2.016 [66]	SH	BA.1, BA.2, BA.2.75, BA.2.75.2, BA.4/5 (453.6–1082 ng/mL)
CC99.103 [85]	SH	BA.1, BA.2, XBB, BA.2.12.1, BA.2.75, BA.2.75.2, BA.4/5, BA.4.6, BQ.1.1 (1.4–20 μg/mL)

NA, not available.
